# GABA_B_ Receptor Modulation of Membrane Excitability in Human Pluripotent Stem Cell‐Derived Sensory Neurons by Baclofen and α‐Conotoxin Vc1.1

**DOI:** 10.1111/jnc.70004

**Published:** 2025-01-27

**Authors:** Mitchell St Clair‐Glover, Arsalan Yousuf, Dominic Kaul, Mirella Dottori, David J. Adams

**Affiliations:** ^1^ Molecular Horizons, Faculty of Science, Medicine and Health University of Wollongong Wollongong New South Wales Australia; ^2^ Sydney Pharmacy School, Faculty of Medicine and Health The University of Sydney Sydney New South Wales Australia

**Keywords:** baclofen, Cav2.2 channel, GABA_B_ receptor, GIRK channel, HCN channel, human sensory neuron, neuroexcitability, α‐conotoxin Vc1.1

## Abstract

GABA_B_ receptor (GABA_B_R) activation is known to alleviate pain by reducing neuronal excitability, primarily through inhibition of high voltage‐activated (HVA) calcium (Ca_V_2.2) channels and potentiating G protein–coupled inwardly rectifying potassium (GIRK) channels. Although the analgesic properties of small molecules and peptides have been primarily tested on isolated murine dorsal root ganglion (DRG) neurons, emerging strategies to develop, study, and characterise human pluripotent stem cell (hPSC)‐derived sensory neurons present a promising alternative. In this study, hPSCs were efficiently differentiated into peripheral DRG‐induced sensory neurons (iSNs) using a combined chemical and transcription factor‐driven approach via a neural crest cell intermediate. Molecular characterisation and transcriptomic analysis confirmed the expression of key DRG markers such as BRN3A, ISLET1, and PRPH, in addition to GABA_B_R and ion channels including Ca_V_2.2 and GIRK1 in iSNs. Functional characterisation of GABA_B_R was conducted using whole‐cell patch clamp electrophysiology, assessing neuronal excitability under current‐clamp conditions in the absence and presence of GABA_B_R agonists baclofen and α‐conotoxin Vc1.1. Both baclofen (100 μM) and Vc1.1 (1 μM) significantly reduced membrane excitability by hyperpolarising the resting membrane potential and increasing the rheobase for action potential firing. In voltage‐clamp mode, baclofen and Vc1.1 inhibited HVA Ca^2+^ channel currents, which were attenuated by the selective GABA_B_R antagonist CGP 55845. However, modulation of GIRK channels by GABA_B_Rs was not observed in the presence of baclofen or Vc1.1, suggesting that functional GIRK1/2 channels were not coupled to GABA_B_Rs in hPSC‐derived iSNs. This study is the first to report GABA_B_R modulation of membrane excitability in iSNs by baclofen and Vc1.1, highlighting their potential as a future model for studying analgesic compounds.

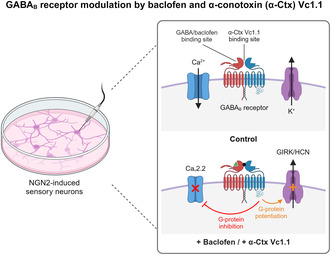

## Graphical abstract

GABA_B_ receptor modulation of membrane excitability in human pluripotent stem cell‐derived sensory neurons by baclofen and α‐conotoxin Vc1.1. Whole‐cell patch‐clamp electrophysiological recording from human NGN2‐induced sensory neurons expressing G protein–coupled GABA_B_ receptors and high voltage‐activated calcium (Cav2.2) channels and inwardly rectifying G protein–coupled potassium (GIRK) and hyperpolarisation‐activated cyclic nucleotide‐gated (HCN) channels. Both baclofen and Vc1.1 significantly reduce neuronal excitability by inhibiting Cav2.2 channels and potentiating GIRK and HCN channel‐mediated currents in induced sensory neurons.

**Abbreviations**
AP: action potential
DEG: differentially expressed gene
DRG: dorsal root ganglion
GABA_B_R: G protein–coupled γ‐aminobutyric acid type B receptor
GIRK: G protein–coupled inwardly rectifying potassium channel
HCN: hyperpolarisation‐activated, cyclic nucleotide‐gated channel
hESC: human embryonic stem cell
hPSC: human pluripotent stem cell
HVA: high voltage‐activated
*I*_Ca_
: Ca^2+^ current
*I*_Kir_
: inwardly rectifying K^+^ current
iSNs: induced sensory neurons
NCC: neural crest cell
NGN2: neurogenin‐2
PRPH: peripheral intermediate filament protein peripherin
RIN: RNA integrity number
RMP: resting membrane potential
TPM: transcripts per million
TPQ: tertiapin‐Q
α‐Ctx: α‐conotoxin

## 1 Introduction

GABA_B_ receptors (GABA_B_Rs) are G protein–coupled receptors for γ‐aminobutyric acid (GABA), the main inhibitory neurotransmitter in the central and peripheral nervous systems. Functional GABA_B_Rs are formed as heteromers of GABA_B1_ and GABA_B2_ subunits, which further associate with various regulatory and signalling proteins to provide receptor complexes with distinct pharmacological and physiological properties (Gassmann and Bettler 2012; Fritzius and Bettler 2020; Fritzius et al. 2022). These receptors play a crucial role in modulating neuronal excitability and synaptic transmission through their effects on various ion channels, including calcium (Ca^2+^) and potassium (K^+^) channels. In sensory neurons, activation of GABA_B_R has been shown to inhibit high voltage‐activated (HVA) Ca^2+^ channels and inwardly rectifying K^+^ channels involved in nociception and pain transmission (Bettler et al. 2004; Padgett and Slesinger 2010; Pinard, Seddik, and Bettler 2010; Benke 2022). By reducing Ca^2+^ influx and increasing K^+^ efflux, GABA_B_Rs contribute to the overall inhibition of neuronal activity, which can dampen pain signals and modulate pain perception.

GABA_B_R play a significant role in modulating pain transmission and perception (Benke 2022; Goudet et al. 2009; Malcangio 2018; Pan et al. 2008; Qian et al. 2023). Activation of GABA_B_R can inhibit pain signalling by modulating membrane ion channels, to regulate neuronal firing properties, and reducing the release of neurotransmitters that transmit pain signals. Specifically, activating GABA_B_R reduces the HVA Ca^2+^ channel currents that control neurotransmitter and hormone release, mediating pain signal transmission in nociceptive neurons (Castro et al. 2017; Klimis et al. 2011; Sadeghi et al. 2017). GABA_B_R activation modulates both HVA (Cav2.2 and Cav2.3) calcium channels (Adams, Callaghan, and Berecki 2012; Berecki et al. 2014; Huynh et al. 2015), and G protein–coupled inwardly rectifying K^+^ (GIRK) channels involved in nociception and pain transmission (Bony et al. 2022; Sadeghi et al. 2017). GABA_B_R functionally couple to GIRK channels to attenuate nociceptive transmission (Blednov et al. 2003), similar to the analgesic effects observed by direct activation of GIRK channels (Abney et al. 2019; Lujan et al. 2014).

Systemic administration of the selective GABA_B_R agonist baclofen has been shown to produce significant analgesic and antinociceptive effects in rodent models of acute and chronic pain, which the intrathecal GABA_B_R antagonists can block. The selective GABA_B_R antagonist CGP35348 can induce mechanical hypersensitivity in naïve rats (Malcangio et al. 1991), whereas intrathecal application of the GABA_B_R agonist baclofen significantly attenuated cancer‐induced bone pain (Zhou et al. 2017). Consistent with the antinociceptive role of GABA_B_Rs, the GABA_B1_ knockout mice lacking GABA binding sites and GABA_B2_ knockout mice, which lack the functional GABA_B_R, both exhibit hyperalgesia (Gassmann et al. 2004; Schuler et al. 2001).

Baclofen and the analgesic α‐conotoxin (α‐Ctx) Vc1.1 have been shown to activate GABA_B_Rs, inhibiting Cav2.2 and Cav2.3 channels and potentiating GIRK‐mediated K^+^ currents in both rodent primary afferent neurons and HEK293T cells expressing human GABA_B_R, Cav2.2/Cav2.3 or GIRK1/2 channels (Berecki et al. 2014; Bony et al. 2022; Cuny et al. 2012). The inhibition of Cav2.2/Cav2.3 channels and potentiation of GIRK channels by Vc1.1 and baclofen via GABA_B_R occurs through a pertussis toxin‐sensitive G protein and can be blocked by the GABA_B_R antagonist CGP 55845. In adult mouse dorsal root ganglion (DRG) neurons, GABA_B_R‐dependent GIRK channel potentiation by Vc1.1 and baclofen hyperpolarizes the cell and reduces its excitability (Bony et al. 2022). However, evidence of this mechanism of action by Vc1.1 and baclofen in human DRG neurons is lacking.

To further elucidate these mechanisms in human tissue, we utilised human pluripotent stem cell (hPSC)‐derived induced sensory neurons (iSNs), generated by overexpressing the neurogenin‐2 (NGN2) transcription factor in neural crest cell (NCC) progenitors. The resulting iSNs were profiled using molecular characterisation and electrophysiological analysis to confirm the expression and function of crucial cell‐specific markers, including the activity of Ca^2+^ and K^+^ channels. This study explored, for the first time, whether GABA_B_Rs in hPSC‐derived iSNs can modulate membrane ion channels to regulate neuronal firing properties similar to that observed in rodents. Overall, we describe fundamental mechanisms involved in the regulation of human sensory neuron excitability, with particular relevance to future studies of novel analgesics.

## 2 Materials and Methods

### 2.1 hPSC Culture

All experiments involving hPSCs were approved by the University of Wollongong Human Research Ethics Committee (HREC 2020/451) and the Institutional Biosafety Committee (Exempt Dealing GT19/08). Briefly, H9^NGN2^ hPSCs derived from WA‐09 (WiCell, Madison, WI) (Miellet et al. 2024) were cultured under feeder‐free conditions using TeSR‐E8 media on Vitronectin XF‐coated plates (STEMCELL Technologies, Vancouver, Canada) at 37°C with 5% CO_2_. Colonies were passaged in bulk at approximately 70% confluence, using 0.5 mM ethylenediaminetetraacetic acid (EDTA) in phosphate‐buffered saline (PBS) for dissociation, and approximately 10% of the cells were reseeded for continued maintenance. Cells were maintained up to a maximum of 20 passages. This cell line is not listed as a commonly misidentified cell line by the International Cell Line Authentication Committee, and further authentication details are available in the associated laboratory resource (Miellet et al. 2024).

### 2.2 Induced Sensory Neuron (iSN) Differentiation

hPSCs were differentiated into iSNs via NCC progenitors following previously published protocols (St Clair‐Glover et al. 2024). Briefly, H9^NGN2^ hPSCs were initially dissociated using Accutase (Sigma‐Aldrich, St. Louis, MO, USA) and seeded onto Matrigel‐coated plasticware (In Vitro Technologies, Melbourne, Australia) at a density of approximately 4.0 × 10^4^ cells/cm^2^. Differentiation into NCCs was performed using the STEMdiff Neural Crest Differentiation Kit (STEMCELL Technologies, Vancouver, Canada), with daily media changes for up to 6 days. NCCs were subsequently harvested using Accutase and enriched via magnetic‐activated cell sorting (MACS, #130–097‐127, Miltenyi Biotec, Germany) to isolate cells expressing the NCC marker CD271. CD271^+^ NCCs were either directly replated for iSN differentiation or cryopreserved in the vapour phase of liquid nitrogen using STEMdiff Neural Induction Medium (STEMCELL Technologies) supplemented with 10% v/v dimethyl sulfoxide (DMSO, Invitrogen, Waltham, MA, USA) as a freezing medium.

In preparation for iSN differentiation, cell culture plasticware was first coated with 10 μg/mL Poly‐D‐Lysine (Sigma‐Aldrich) for 30 min at room temperature, followed by coating with Matrigel overnight at 37°C. CD271^+^ NCCs were seeded at densities of approximately 8.0 × 10^3^ cells/cm^2^ on 12 mm circular glass coverslips for patch‐clamp studies, 2.6 × 10^4^ cells/cm^2^ on 13 mm circular glass coverslips for immunocytochemistry, or 2.6 × 10^4^ cells/cm^2^ directly on plasticware for transcriptomic analyses. iSN differentiation was carried out following previously published protocols with modifications (Hulme et al. 2020; St Clair‐Glover et al. 2024). Briefly, cells were cultured in neuronal medium comprising Neurobasal medium supplemented with 1% N_2_, 1% B‐27 minus vitamin A, 1% Insulin‐transferrin‐Selenium‐A, and 1% GlutaMAX (all from Gibco, Waltham, MA, USA). During iSN differentiation, the medium was further enriched with 10 μM Y‐27632, 10 ng/mL BDNF, 10 ng/mL GDNF, 10 ng/mL NT‐3, and 10 ng/mL β‐NGF (all from STEMCELL Technologies), with media changes every 2–3 days. For the first 96‐h post‐plating (days 6–10), NGN2 expression was induced by the application of 2 μg/mL doxycycline (Sigma‐Aldrich, D9891). Starting on day 14, cells were transitioned into BrainPhys neuronal medium (BrainPhys Neuronal Medium, 0.02% NeuroCult SM1 without Vitamin A, 0.01% N2 Supplement‐A [all from STEMCELL Technologies]) for maturation, using a stepwise a ratio of BrainPhys: neuronal medium (25:75, 50:50, 75:25, 100:0) at each media change. To inhibit proliferating cells, 2.5 μM cytosine β‐D‐arabinofuranoside (AraC, Sigma‐Aldrich), an antimitotic agent, was added for 48 h between days 16 and 18. iSNs were cultured for approximately 27 days, after which they were either fixed for immunocytochemistry or harvested for RNA analysis. For functional studies, iSNs were used for patch‐clamp recordings between days 24 and 27.

### 2.3 Immunocytochemistry

H9^NGN2^ hPSCs, NCCs, and iSNs were cultured on 13 mm circular glass coverslips for immunocytochemistry analysis. In preparation for staining, cells were first fixed with 4% w/v paraformaldehyde in PBS (Sigma‐Aldrich) for 15 min, permeabilised with 0.3% v/v Triton X‐100 in PBS for 10 min, and then blocked for 1 h using 10% v/v normal donkey serum in PBS (Sigma‐Aldrich). Primary antibody solutions were applied overnight at 4°C, followed by washing and incubation for 1 h with secondary antibodies and a 2 μg/mL DAPI nuclear counterstain (D9542, Sigma‐Aldrich). Coverslips were mounted on glass slides with ProLong Glass antifade mountant (Invitrogen) and imaged using a Leica SP8 confocal microscope system on a 20× dry objective or 40×, 63×, or 92× oil‐immersion objective. Acquired images were then exported and analysed using the Fiji software package (Schindelin et al. 2012).

Antibodies used for immunocytochemistry were those against SOX2 (AF2018; R&D Systems), OCT4 (sc‐5279 Santa Cruz Biotechnology, Dallas, TX, USA), CD271 (M‐1818‐100; Biosensis, Thebarton, SA, Australia), SOX10 (AF2684; R&D Systems, Minneapolis, MN, USA), BRN3A (MAB1585; Millipore, St. Louis, MO, USA), ISLET1 (ab20670; Abcam, Cambridge, UK), TUJ1 (MAB1637, Millipore), TUJ1 (ab18207, Abcam), Peripherin (ab4666, Abcam), NGN1 (MA5‐24900, Invitrogen), NGN2 (PA5‐78556, Invitrogen), GABBR1 (AP23115PU‐N; OriGene, Rockville, MD, USA), GABBR2 (ab75838, Abcam), Ca_V_2.2 (KP10001, CALBIOCHEM), GIRK1 (APC‐005; Alomone Labs, Jerusalem, Israel), Na_V_1.7 (ab65167, Abcam), Na_V_1.8 (ab66743, Abcam), S100β (ab52642, Abcam), MAP2 (M4403, Sigma‐Aldrich), and PSD95 (#51–6900, Invitrogen). Secondary antibodies were those against mouse (ab150109, Abcam), rabbit (ab150062, Abcam) and goat (ab150135, Abcam) IgG.

### 2.4 Bulk RNAseq

Total RNA extraction was performed on iSNs using the PureLink RNA Mini Kit (Invitrogen) following the manufacturer's directions (*n* = 3 independent cell culture preparations). Pure RNA libraries (RNA integrity number (RIN) ≥ 7) were then sequenced at the Kinghorn Centre for Clinical Genomics (Garvan Institute of Medical Research, Australia) using the NextSeq 550Dx platform (Illumina, San Diego, CA, USA), aiming for > 100M single reads per sample (total number of alignments 54M–63M, v2.5 chemistry). Resulting FASTQ files were aligned with the human genome (genome build GRCh38.109) using the nf‐core/RNAseq pipeline (Ewels et al. 2020). Briefly, FASTQ files were pre‐processed to infer strandedness (Salmon), trim reads (Trim Galore!), and remove genome/ribosomal contamination (BBSplit and SortMeRNA, respectively). Reads were then aligned to reference genome using STAR and Salmon (Dobin et al. 2013; Patro et al. 2017). Quality control of read alignment was conducted using MulitQC, qualimap and FastQC. Transcripts per million (TPM) were calculated using the cpm function in edgeR (version 3.17, R). Publicly available data for single nucleus RNA sequencing for human DRG (Jung et al. 2023) was downloaded and pseudobulking performed using ADPBulk (version 0.1.4, Python) to extract the sum of raw UMI counts. Raw counts were then processed according to the same cpm function. To allow for comparison of specific genes of interest, cpm for curated genes were scaled between 0 and 1 for the human DRG snRNAseq dataset and the iSN dataset. Datasets were compared using Pearson's correlations on scaled cpm values. A confidence interval of the correlation coefficient was reported.

### 2.5 Differential Expression and Gene Set Enrichment Analysis

Differentially expressed genes (DEGs) between iSNs and a publicly available H9 embryonic stem cell RNAseq dataset (Chu et al. 2016) were determined using DESeq2 (version 1.38.3, R), according to standard parameters. Significant DEGs were defined using a threshold of abs[log_2_FoldChange] > 1, *P*_FDR_ < 0.05.

Gene set enrichment analysis was performed on DEGs using fGSEA (version 1.25.2, R). The molecular function and cellular compartment gene sets were downloaded from msigdb (msigdbr version 7.5.1, R). Gene rank was determined by (−sign(log_10_(*P*)) × log_2_FoldChange), and gene set size was set between 15 and 501.

### 2.6 Whole‐Cell Patch‐Clamp Electrophysiology

Whole‐cell patch‐clamp recordings of iSNs were made at room temperature (21°C–23°C) using a MultiClamp 700B Amplifier and signals were digitalized with a Digidata 1440 and controlled with pClamp11 software (Molecular Devices, San Jose, CA, USA). In whole‐cell experiments, electrical access was established either by rupturing the membrane patch and dialysing the cell, or by using the perforated patch method. For perforated patch experiments, a stock solution of 50 mg/mL of amphotericin B in DMSO was prepared on the day of the experiment. Just prior to use, the amphotericin B stock solution was diluted in pipette solution to yield a final concentration of 240 μg/mL amphotericin B in 0.4% DMSO. Whole‐cell membrane currents were sampled at 100 kHz, filtered at 10 kHz, and series resistance compensated 70%–80%. Fire‐polished borosilicate (1B150F‐4, World Precision Instruments, USA) patch pipettes were used with resistance 2–4 MΩ and filled with an intracellular solution containing (in mM): 140 K‐Gluconate, 10 NaCl, 2 MgCl_2_, 5 EGTA (ethylene glycol‐bis(β‐aminoethyl ether)‐N,N,N′,N′‐tetraacetic acid) and 10 HEPES (2‐[4‐(2‐hydroxyethyl)piperazin‐1‐yl]ethanesulfonic acid), pH 7.2. Liquid junction potential was calculated to be +16 mV and was corrected before each experiment.

The resting membrane potential (RMP) was recorded immediately after switching into the current‐clamp mode as the average membrane voltage in the absence of current injection. Membrane potentials were adjusted by injecting increasing bias currents. Action potential (AP) frequency was calculated by challenging the hPSC‐derived iSNs with increasing increments of current injections, each lasting for 500 msec. The firing frequency was assessed by counting the number of APs fired during these current injections, both in the absence and presence of drugs. The data were plotted using GraphPad Prism 7 (GraphPad Software, San Diego, CA, USA) and subjected to further statistical analysis. The rheobase, or current threshold, was defined as the minimum amount of current necessary to evoke a single AP during 500 ms depolarizing current steps in 5 pA increments. Elicited APs were counted and plotted as a function of the current injection intensity during stimulation. For all current‐clamp experiments, the perforated patch‐clamp method was used, with an extracellular solution with the following composition (in mM) was used: 135 NaCl, 2 CaCl_2_, 2 MgCl_2_, 5 KCl, 10 D‐Glucose, 10 HEPES and pH 7.3. Both perforated patch‐clamp and dialysed whole‐cell recording were used for voltage‐clamp experiments, with the extracellular solution adjusted based on the ion channel being examined. To study hyperpolarisation‐activated K^+^ currents (*I*_K_), the K^+^ concentration of the extracellular solution above was raised to 20 mM. To isolate depolarization‐activated Ca^2+^ currents (*I*_Ca_), the extracellular solution contained (in mM): 140 TEA‐Cl, 10 CaCl_2_, 1 MgCl_2_, 10 HEPES, 10 D‐Glucose and pH 7.3. In a series of experiments, the extracellular solution was supplemented with 1 μM tetrodotoxin citrate (TTX; Alomone Labs). No significant differences were observed between perforated patch and dialyzed whole‐cell experiments. The following drugs were applied by bath perfusion at the concentrations stated in the Results: (±)‐β‐(aminomethyl)‐4‐chlorobenzenepropanoic acid (baclofen), GABA, and Pertussis toxin (PTX) were purchased from Sigma‐Aldrich. 4‐Ethylphenylamino‐1,2‐dimethyl‐6‐methylaminopyrimidinium chloride (ZD7288) and CGP 55845 ((2S)‐3‐[[(1S)‐1‐(3,4‐dichlorophenyl)ethyl]amino‐2‐hydroxypropyl](phenylmethyl) phosphinic acid hydrochloride) were purchased from Tocris Bioscience (Bristol, UK). Tertiapin‐Q (TPQ) was purchased from Abcam. Synthetic α‐conotoxin Vc1.1 was supplied by Dr. Richard Clark (The University of Queensland, Brisbane, QLD, Australia). All drugs were dissolved in distilled H_2_O to prepare to the appropriate stock concentration, except CGP 55845, which was dissolved in DMSO. The final concentration of DMSO did not exceed 0.01%.

### 2.7 HEK293T Cell Culture, Transfection and Whole‐Cell Voltage‐Clamp Recording

HEK293T cells expressing the SV40 large T‐antigen (ATCC CRL‐3216, RRID‐CVCL_0063) were cultured in Dulbecco's modified Eagle's medium (DMEM GIBCOTM, Cat # 21331–020; Thermo Fisher Scientific). This cell line is not classified as a commonly misidentified cell line by the International Cell Line Authentication Committee, and further authentication was performed Short Tandem Repeat (STR) profiling at the Garvan Institute of Medical Research, Australia. HEK293T cells were also supplemented with 10% FBS (Bovigen, Australia), 1% penicillin and streptomycin and 1× GlutaMAX (Cat # 35050–061; Thermo Fisher Scientific) at 37°C in 5% CO_2_. The cells were passaged at approximately 80% confluency following standard procedures and maintained up to a maximum of 20 passages. For transfection, cells were plated on 12 mm glass coverslips and transiently transfected using the calcium phosphate method. Plasmid cDNAs encoding the human GABA_B1_ and GABA_B2_ subunits were obtained from OriGene Technologies Inc., (Rockville, MD USA) and the human HCN1 and two clones were provided by Dr. Christopher Reid (Florey Institute, Melbourne, Australia) A green fluorescent protein (GFP) expressing plasmid was also used to identify transfected cells.

Whole‐cell patch‐clamp recordings were performed within 48 h post‐transfection at room temperature (21°C–23°C). A ‘high K^+^’ external bath solution was used to record hyperpolarisation‐activated currents and contained (in mM): 140 TEA‐Cl, 20 KCl, 2 CaCl_2_, 1 MgCl_2_, 10 Glucose, 10 HEPES (pH 7.35 with TEA‐OH; ~310 mOsmol·kg^−1^). Fire‐polished borosilicate pipettes (1–3 MΩ) were filled with intracellular solution containing (in mM): 140 K‐Gluconate, 5 NaCl, 2 MgCl_2_, 5 EGTA, 10 HEPES (pH 7.2 with KOH; ~290 mOsmol·kg^−1^). Hyperpolarisation‐activated currents were elicited by hyperpolarising the cells to −100 mV (3 s duration) from a holding potential of −40 mV. In a series of experiments, inhibition of heterotrimeric G_i/o_ proteins was achieved by 24 h incubating the transfected cells with 0.1 μg/mL PTX before recording the hyperpolarisation‐activated currents.

### 2.8 Data Analysis and Statistics

All data represent mean ± SD unless otherwise noted. No exclusion criteria were pre‐determined, and randomisation was not applied when assigning treatments. Sample size calculations were not performed, and neither blinding nor outlier testing was conducted. Paired comparisons of current‐clamp and voltage‐clamp parameters were performed using Wilcoxon signed‐rank test unless otherwise specified. A Gaussian distribution was assumed for all data; however, normality was not assessed, therefore, non‐parametric tests were used for all multiple group comparisons as required. All statistical tests were two‐tailed. *N* indicates the number of independent observations, and asterisks indicate statistical significance: *p < 0.05, **p < 0.005 and ***p < 0.001.

Clampfit 11.0 (Molecular Devices) and GraphPad Prism 7 software were used for data analysis. All data represent mean ± SD unless stated otherwise. For current‐clamp or excitability parameters analysis, iSNs with RMPs more negative than −40 mV were accepted. Membrane input resistance was calculated from a series of nine small hyperpolarising current pulses from 0 to −40 mV by calculating the slope of the linear fit in the absence and presence of baclofen and α‐Vc1.1. Numbers of AP were calculated at a current injection of 2× rheobase, in both control and after application of peptide. Rheobase threshold potential was determined at which evoked the first AP either in control condition or after the application of peptide.

For GABA_B_R‐Cav2.2 measurements, all data points are plotted as % inhibition using the following equation:

$$I_{\mathrm{Ca}}\;\text{inhibition}\;\left(\%\right)=\left(I_{\text{Ctrl}}—I_{\mathrm{Bac}}/I_{\text{Ctrl}}\right)\times 100$$

Analysis of peak inwardly rectifying K^+^ currents (*I*_Kir_) in the presence of GIRK channel inhibitor (Tertiapin‐Q) and hyperpolarisation‐activated, cyclic nucleotide‐gated (HCN) channel inhibitor (ZD7288) was performed using the following equation:

$$\%I_{\mathrm{Kir}}=\frac{I_{K\left(\text{Ctrl}\right)}-I_{K\left(\mathrm{ZD}7288\right)}}{I_{K\left(\text{Ctrl}\right)}}\times 100$$

## 3 Results

### 3.1 Generation of hPSC‐Derived iSNs via Inducible NGN2 Expression

**FIGURE 1:**
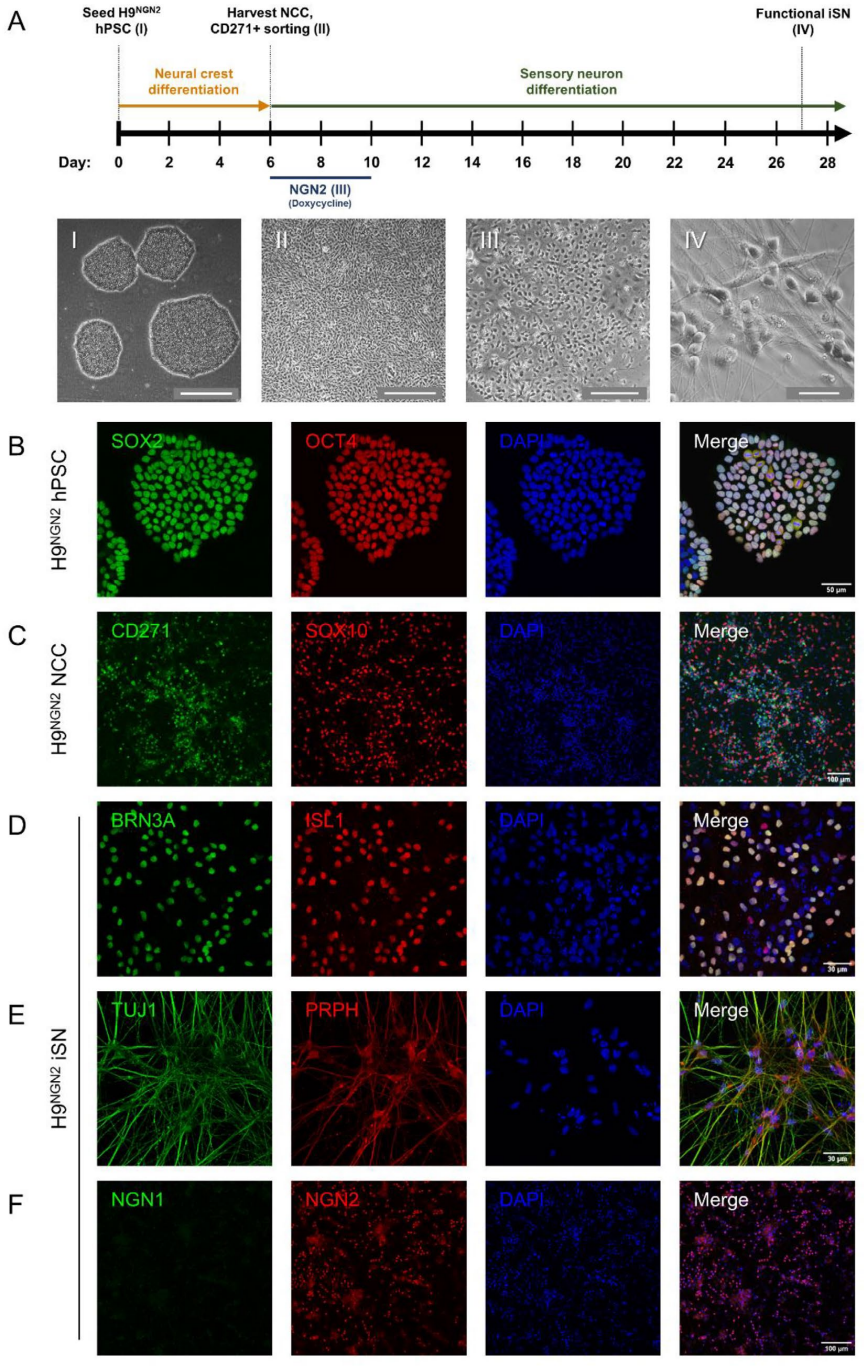
Generation of H9^NGN2^ human pluripotent stem cell (hPSC)‐derived induced sensory neurons (iSNs). (A) Schematic protocol for the differentiation of iSNs. Briefly, hPSC are differentiated to neural crest cells (NCCs, days 1–6), then enriched for the cell surface marker CD271 via magnetic cell sorting. CD271^+^ NCCs are plated for sensory neuron differentiation (days 6–27), with induction of NGN2 expression via application of doxycycline for 96 h (days 6–10). (A) (I–IV) Representative micrographs of H9^NGN2^ through differentiation to iSNs; (I) hPSC colonies, scale bar = 500 μm; (II) NCC after 6 days differentiation, scale bar = 500 μm; (III) 96 h NGN2‐induced neurite outgrowth, scale bar = 200 μm; (IV) iSNs after 3 weeks differentiation and maturation, scale bar = 50 μm. (B–F). Immunocytochemistry staining for molecular markers through hPSC differentiation to iSNs. (B) H9^NGN2^ hPSC stained for pluripotency transcription factors SOX2 (green) and OCT4 (red). (C) H9^NGN2^‐derived NCC stained for cell surface receptor CD271 (green) and transcription factor SOX10 (red). (D) iSNs stained for BRN3A (green) and ISL1 (red) transcription factors. (E) iSNs stained for neural marker TUJ1 (green) and peripheral neuronal marker PRPH (red). (F) iSNs stained for neurogenesis transcription factors NGN1 (green) and NGN2 (red).

The efficient and accelerated differentiation of neurons from hPSCs has been robustly demonstrated through forced expression of the NGN2 transcription factor (Hulme et al. 2022). This study utilised a transgenic H9^NGN2^ hPSC line containing a doxycycline‐inducible NGN2 gene expression cassette for the generation of iSNs (Miellet et al. 2024). We adapted our previously established protocols to generate iSNs via an NCC progenitor stage, with overexpression of NGN2 to drive sensory neurogenesis (Hulme et al. 2020; St Clair‐Glover et al. 2024). In this approach, H9^NGN2^ hPSCs were first differentiated to NCC over 6 days in a monolayer, then over a further 21 days were differentiated to iSNs (Figure 1A). Maintenance colonies of the H9^NGN2^ hPSCs exhibited co‐expression of the transcription factors SOX2 and OCT4, hallmark indicators of pluripotency (Figure 1AI,B). Following 6 days of differentiation, a distinct epithelial to mesenchymal morphology change was associated with expression of the markers SOX10 and CD271 in NCCs (Figure 1AII,C; Figure S1). NCCs were then differentiated to iSNs over a further 21 days, with an initial 96 h doxycycline application to induce NGN2 expression (Figure 1AIII). iSN differentiation was evidenced by a neuronal morphology change and extensive neurite outgrowth, with robust co‐expression of the sensory neuron transcription factors BRN3A and ISLET1 (Figure 1AIV,D). Further immunocytochemistry staining revealed expression of the neuronal markers βIII‐tubulin (TUJ1), peripheral intermediate filament protein peripherin (PRPH), and the NGN2 transcription factor (Figure 1E,F), consistent with sensory neuron differentiation. The iSNs did not express NGN1, highlighting the specificity of the NGN2 gene expression cassette in driving sensory neurogenesis (Figure 1F).

### 3.2 Transcriptomic Characterisation of iSNs

**FIGURE 2:**
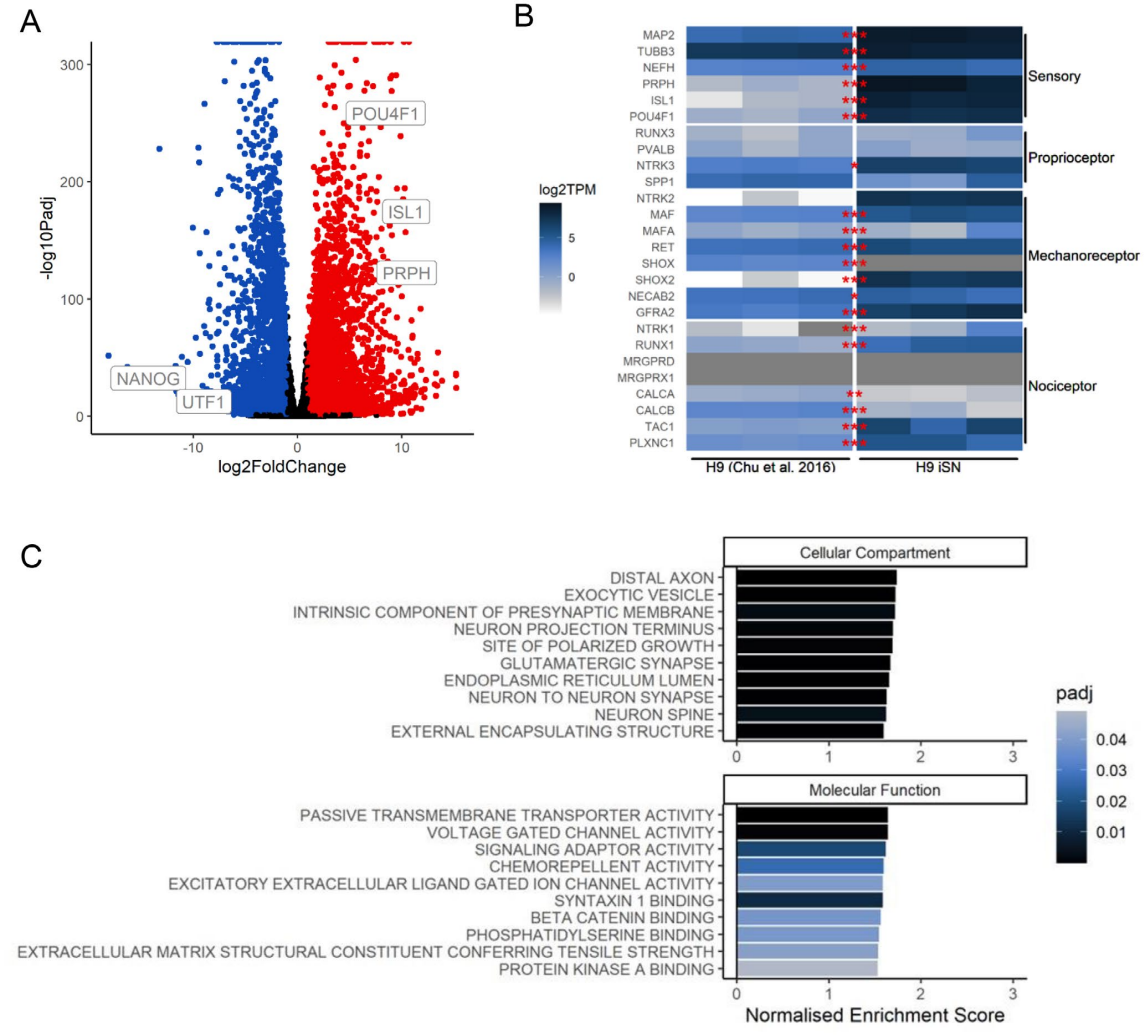
Bulk RNA sequencing analysis of H9^NGN2^‐induced sensory neurons (iSNs) reveals transcriptomic expression of sensory neuron markers. (A) Volcano plot showing differentially expressed genes in iSN relative to H9 embryonic stem cells (hESCs). Significance thresholds are |logfc| > 1 and *P*_adj_ < 0.05. Red dots indicate genes upregulated in iSNs, and blue dots indicate those downregulated. (B) Heat map comparing normalised expression levels (colour bars, log_2_TPM) of curated sensory neuron genes (rows) between H9 hESCs (Chu et al. 2016) and H9^NGN2^ iSNs. Grey denotes no transcripts were detected by sequencing. Significantly upregulated genes are denoted as *P*_adj_ < 0.001***, < 0.01**, < 0.05*. (C) Gene set enrichment analysis (GSEA) of H9^NGN2^ iSNs for cellular compartment (top) and molecular function (bottom) using gene ontology databases. Rank = sign(logfc) × −log_10_ *P*. Bars represent the 10 most enriched functions (normalised enrichment score) for each gene set, with colour denoting adjusted *p*‐value (*P*_adj_ < 0.5), pruning for semantic similarity.

To further characterise this model, we performed bulk RNA sequencing to assess the transcriptome of H9^NGN2^‐derived iSNs. Differential expression analysis between H9^NGN2^ iSNs and H9 human embryonic stem cells (hESCs; Chu et al. 2016) confirmed a significant (*P*_adj_ < 0.05) increase in 5796 genes, including sensory neuron markers (*PRPH*, *POU4F1,* and *ISL1*) and a decrease in 6036 genes, including proliferative genes (*NANOG* and *UTF1*; Figure 2A). Sensory neuron marker expression was consistently increased between biological replicates (Figure 2B). Mapping expression onto curated gene sets of SN subtypes (proprioceptive, mechanoreceptive, and nociceptive neurons) showed expression of markers across all three sensory neuron subtypes, indicating a mixed population of sensory neurons in iSN cultures (Figure 2B).

To determine common functions increased in iSNs, gene set enrichment analysis was performed using the cellular compartment and molecular function gene ontology databases. Relative to H9 ESCs, iSNs demonstrated a significant enrichment in both neuronal functions (e.g., voltage‐gated channel activity [*P*_adj_ = 2.02 × 10^−6^]) and mature neuronal cellular structures (e.g., Distal Axon [*P*_adj_ = 7.93 × 10^−6^], Neuron to Neuron Synapse [*P*_adj_ = 7.93 × 10^−6^]) (Figure 2C). The delineation of H9 hPSCs and H9^NGN2^‐derived iSNs through transcriptomic analysis therefore suggests that the molecular profile of iSNs is generally consistent with native human SNs.

### 3.3 iSNs Express GABA_B_R, Ca^2+^ and K^+^ Channels Which Contribute to Membrane Excitability

**FIGURE 3:**
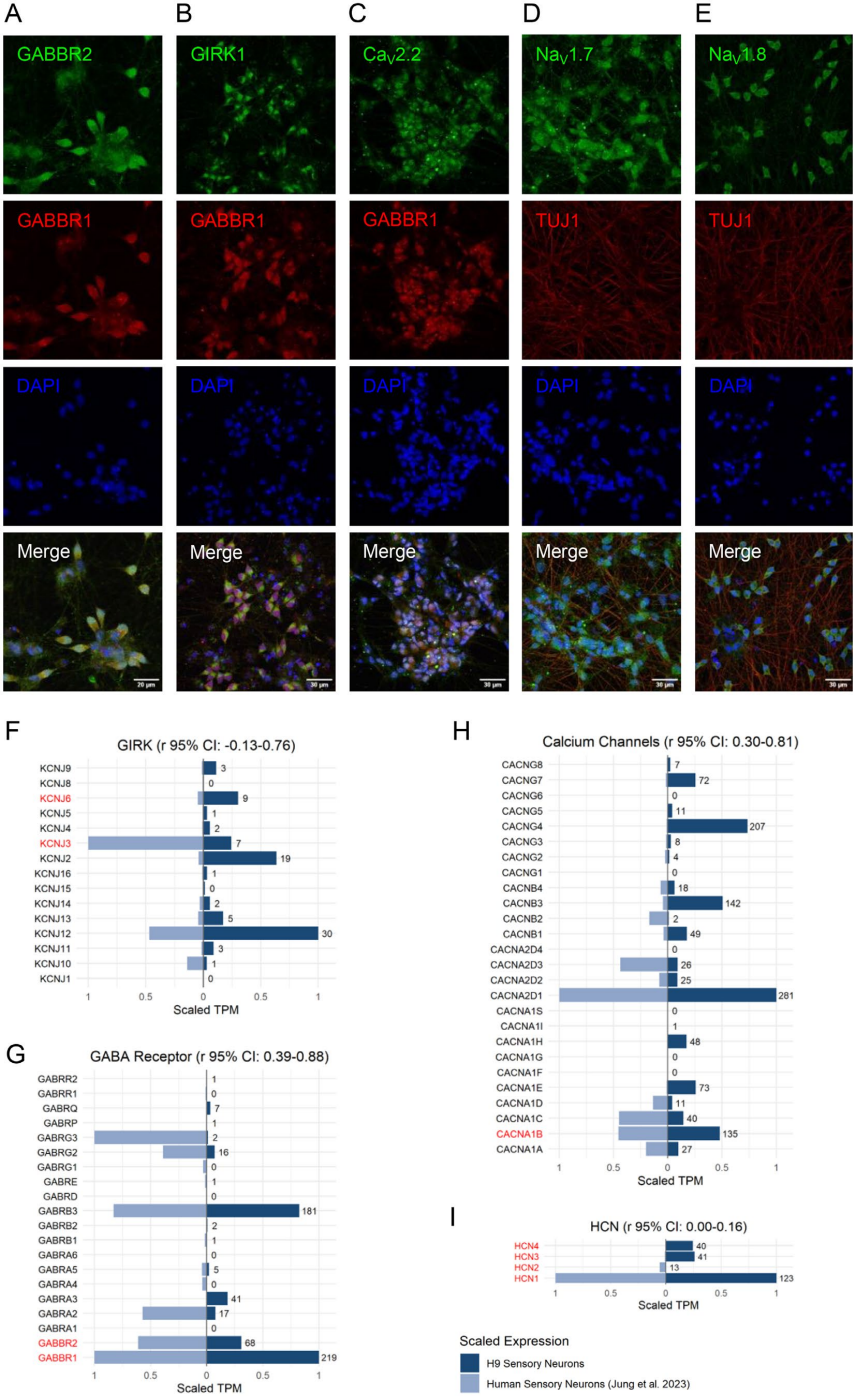
Expression of ion channels and membrane proteins in H9^NGN2^‐induced sensory neurons (iSNs) that modulate neuronal excitability. (A–E) Immunocytochemistry staining in H9^NGN2^ iSNs: (A) GABA_B_ receptor subunits GABBR2 (green) and GABBR1 (red). (B) G protein–coupled inwardly rectifying potassium channel subunit 1 (GIRK1/KCNJ3, green) and GABBR1 (red). (C) Voltage‐gated calcium channel (Ca_V_2.2/CACNA1B, green) and GABBR1 (red). (D) Voltage‐gated sodium channel (Na_V_1.7/SCN9A, green) and neuronal marker TUJ1 (red). (E) Voltage‐gated sodium channel (Na_V_1.8/SCN10A, green) and neuronal marker TUJ1 (red). (F–I) Scaled transcriptomic expression (TPM) of curated genes in discrete classes of ion channels and membrane receptors. Expression levels in iSNs (dark blue, TPM values denoted) are compared to primary human sensory neurons (light blue), with genes pertinent to this study highlighted in red. (F) G protein–coupled inwardly rectifying K^+^ channel (GIRK) subunit expression. (G) GABA_B_ receptor subunit expression. (H) Calcium channel subunit expression. (I) Hyperpolarisation‐activated, cyclic nucleotide‐gated (HCN) channel subunit expression.

Given that fundamental properties of neuronal excitability are underpinned by membrane receptors and ion channels, we sought to interrogate their expression patterns in iSNs. Immunocytochemistry analysis revealed expression of the voltage‐gated ion channels Ca_V_2.2, Na_V_1.7, Na_V_1.8, and G protein–coupled inwardly rectifying potassium channel subunit 1 (GIRK1) in iSNs (Figure 3A,C–E). Further, the G protein–coupled receptor subunits GABBR1 and GABBR2 appeared co‐expressed in the iSN membrane (Figure 3A,B).

Bioinformatics analysis confirmed that these proteins were expressed at the transcriptomic level. This included genes encoding GIRK1 (*KCNJ3*), GIRK2 (*KCNJ6*), *GABBR1*, *GABBR2,* and HCN subunits 1–4, which are highly relevant to the electrophysiological properties investigated in this study (Figure 3F,G,I). Comparing the scaled proportions of these genes in native human DRG, we found a positive correlation in the Ca^2+^ channels and GABA_B_R (95% CI 0.30–0.81 and 0.39–0.88, respectively) with no significant correlation in GIRK and HCN channels (95% CI −0.13 to 0.76 and 0.00 to 0.16, respectively) (Figure 3F–I). Together, this expression data suggests that iSNs possess the molecular machinery that underpins critical membrane excitability properties.

### 3.4 GABA_B_R Agonists Affect Membrane Excitability in iSNs

**FIGURE 4:**
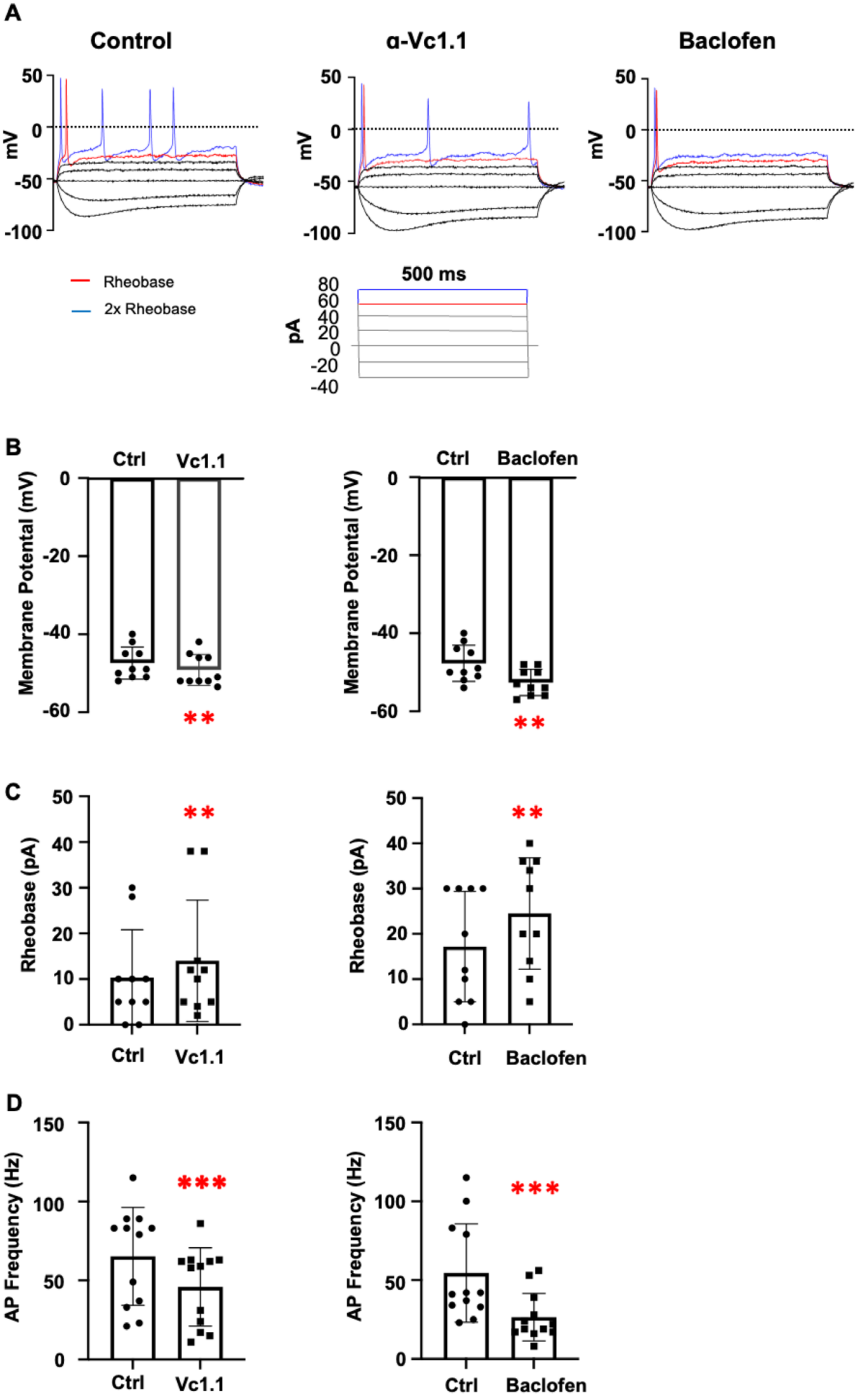
α‐Conotoxin Vc1.1 hyperpolarizes the resting membrane potential (RMP) and reduces excitability in H9^NGN2^‐induced sensory neurons (iSNs), similar to the canonical GABA_B_R agonist baclofen. (A) Representative voltage responses to current‐clamp steps recorded from an iSN in the absence (control; left) and presence of 1 μM Vc1.1 (middle), or 100 μM baclofen (right). The dashed line indicates 0 mV; red indicates the rheobase, and blue indicates 2× rheobase for both membrane potential and current. (B–D) Bar graphs and scatter plots showing the effects of 1 μM Vc1.1 and 100 μM baclofen on (B) RMP (Vc1.1, *n* = 10, **p = 0.0039; baclofen, *n* = 10, *p = 0.0020), (C) rheobase (Vc1.1, *n* = 10, **p = 0.0078; baclofen, *n* = 10, **p = 0.0039), and (D) AP frequency in response to 500 ms depolarizing current steps (Vc1.1, *n* = 12, ***p = 0.0005; baclofen, *n* = 12, ***p = 0.0005). Data are presented as mean ± SD; paired Wilcoxon signed‐rank test. *N* = 10–12 for each column.

Our previous studies of hPSC‐derived sensory neurons indicate the functional expression of various ion channels (Na^+^, K^+^, Ca^2+^ and Cl^−^) that regulate membrane excitability (Hulme et al. 2020, 2024; St Clair‐Glover et al. 2024). Additionally, the RMP of iSNs is comparable to that observed in mammalian sensory neurons (Rasband et al. 2001; Hulme et al. 2020). Given our identification of GABA_B_R expression at both the transcript and protein level in iSNs (Figure 3), we aimed to investigate the function of these receptors. Whole‐cell patch‐clamp electrophysiology was used to assess the functional expression of GABA_B_Rs in small to medium diameter (< 30 μm) iSNs. The passive and active membrane properties of iSNs were recorded both in the absence and presence of the GABA_B_R agonists baclofen and α‐Ctx Vc1.1, using gravity flow bath perfusion. Both α‐Ctx Vc1.1 and baclofen significantly affected membrane excitability parameters (Figure 4). Specifically, bath application of 1 μM Vc1.1 hyperpolarised the RMP from −47.4 ± 4.0 mV (control) to −49.0 ± 4.0 mV (*n* = 10, *p* = 0.0039) and to −52.6 ± 3.0 mV (*n* = 10, *p* = 0.0020) in the presence of 100 μM baclofen (Figure 4B). Vc1.1 (1 μM) increased the rheobase for AP firing from 10 pA (control) to 14 pA (*n* = 10, *p* = 0.0078), while baclofen (100 μM) increased the rheobase from 17 pA (control) to 24.5 pA (*n* = 10, *p* = 0.0039) (Figure 4C). The elevated rheobase caused a decrease in AP firing in response to depolarizing current pulses in the presence of Vc1.1 (*n* = 12, *p* = 0.0005) and baclofen (*n* = 12, *p* = 0.0005) (Figure 4D). No significant difference was observed in membrane input resistance for either Vc1.1 (*n* = 9, *p* = 0.357) or baclofen (*n* = 9, *p* = 0.1759).

**FIGURE 5:**
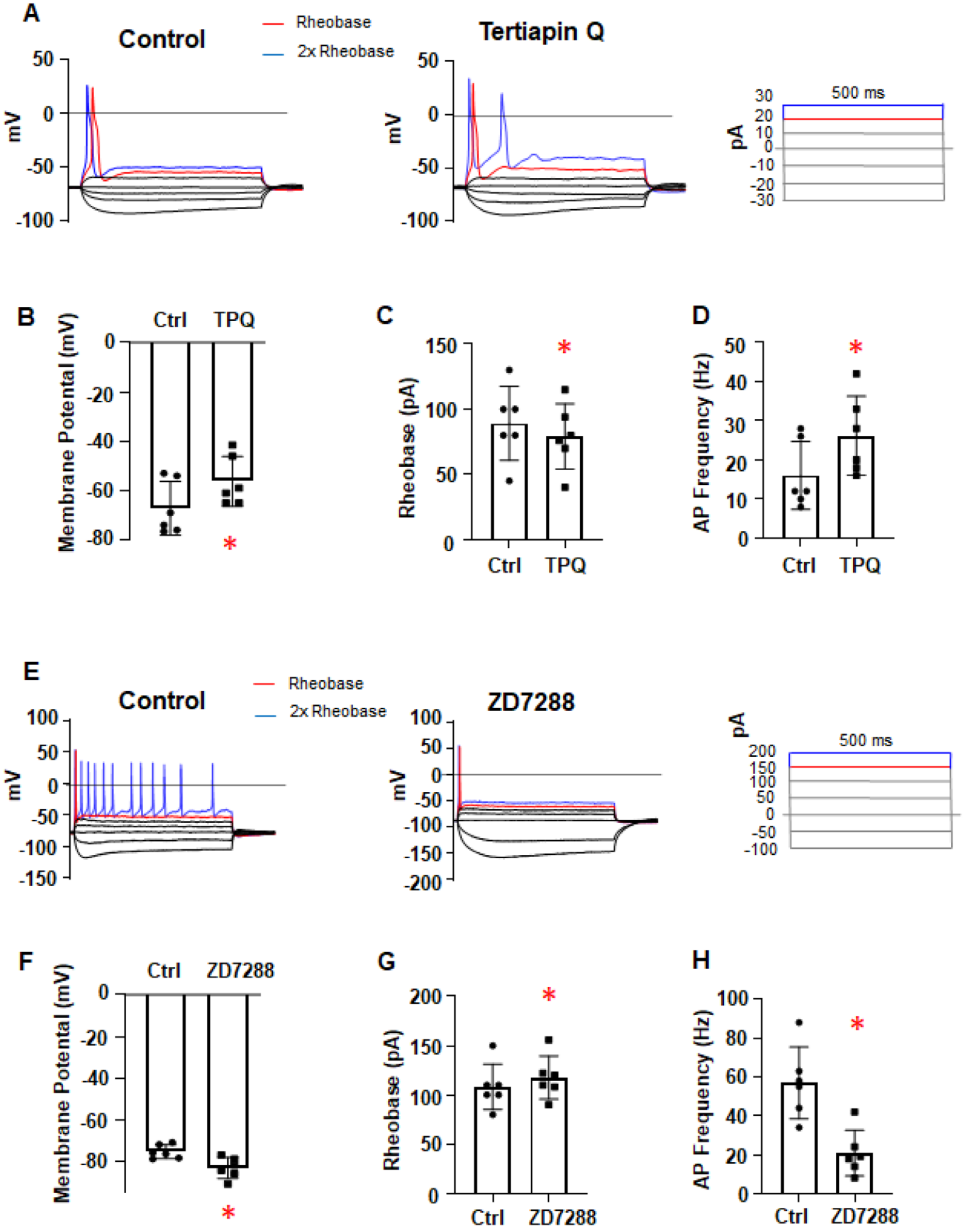
Effects of the GIRK channel inhibitor, Tertiapin‐Q, and HCN channel inhibitor, ZD7288, on membrane excitability parameters in H9^NGN2^‐induced sensory neurons (iSNs). (A, E) Representative voltage responses to current‐clamp steps recorded from iSNs in the absence (control; left) and presence of 100 nM TPQ (A) or 30 μM ZD7288 (E). The dashed line indicates 0 mV; red denotes the rheobase, and blue denotes 2× rheobase for both membrane potential and current. (B–D and F–H) Bar graphs and scatter plots showing the effects of 100 nM TPQ (B–D) and 30 μM ZD7288 (F–H) on (B, F) resting membrane potential (RMP) (*p = 0.0312), (C, G) rheobase (*p = 0.0312), and (D, H) action potential (AP) frequency (*p = 0.0312). Data are presented as mean ± SD; paired Wilcoxon signed‐rank test. *N* = 6 for each condition.

We also investigated the functionality of hyperpolarisation‐activated cation channels, such as GIRK and HCN channels, expressed in mammalian central and peripheral neurons. When iSNs were exposed to the GIRK inhibitor tertiapin‐Q (100 nM) and the HCN inhibitor ZD7288 (30 μM), both passive and active membrane properties were significantly altered. Bath application of tertiapin‐Q depolarized the RMP by 11 ± 2 mV, whereas ZD7288 hyperpolarized the RMP by 8 ± 3 mV (*n* = 6, *p* = 0.0313) (Figure 5B,F). Additionally, tertiapin‐Q (100 nM) reduced the rheobase by 11 ± 7 pA and approximately doubled the AP firing frequency (*n* = 6, *p* = 0.0313) (Figure 5C,D). In contrast, ZD7288 (30 μM) increased the rheobase by 9 ± 2 pA and reduced AP firing frequency by approximately three‐fold (*n* = 6, *p* = 0.0313) (Figure 5G,H). Notably, the characteristic ‘hump’ or depolarizing sag observed in the hyperpolarisation‐activated voltage responses mediated by HCN channel currents (*I*_h_) remained largely unaffected by the GABA_B_R agonists Vc1.1 or baclofen (Figure 4A). However, this HCN channel‐mediated depolarizing sag, observed under control conditions, was completely abolished by the specific HCN inhibitor ZD7288 (Figure 5E).

Taken together, these results demonstrate that the GABA_B_R agonists baclofen and Vc1.1 modulate hyperpolarisation‐activated K^+^ channels and alter membrane excitability properties in human iSNs in a manner consistent with previous reports in mouse DRG neurons (Bony et al. 2022; Yousuf et al. 2022).

### 3.5 GABA_B_R Agonists Inhibit Baclofen‐Sensitive HVA Ca^2+^ Channels in iSNs

A key factor in controlling neuronal excitability at central and peripheral synapses is the modulation of voltage‐gated Ca^2+^ and GIRK channels (Pan et al. 2008; Waxman and Zamponi 2014). GABA_B_R has been shown to influence neuronal excitability by inhibiting HVA Ca^2+^ channels and potentiating GIRK channels in rodent DRG neurons (Bony et al. 2022; Yousuf et al. 2022). Given that GABA_B_R agonists baclofen and α‐Ctx Vc1.1 were found to affect membrane firing properties in iSNs, we sought to further confirm their interaction with HVA Ca^2+^ channels. Using whole‐cell patch‐clamp electrophysiology under voltage‐clamp configuration, we tested the effects of 1 μM Vc1.1 and 50 μM baclofen using a step depolarization from −80 to 0 mV.

**FIGURE 6:**
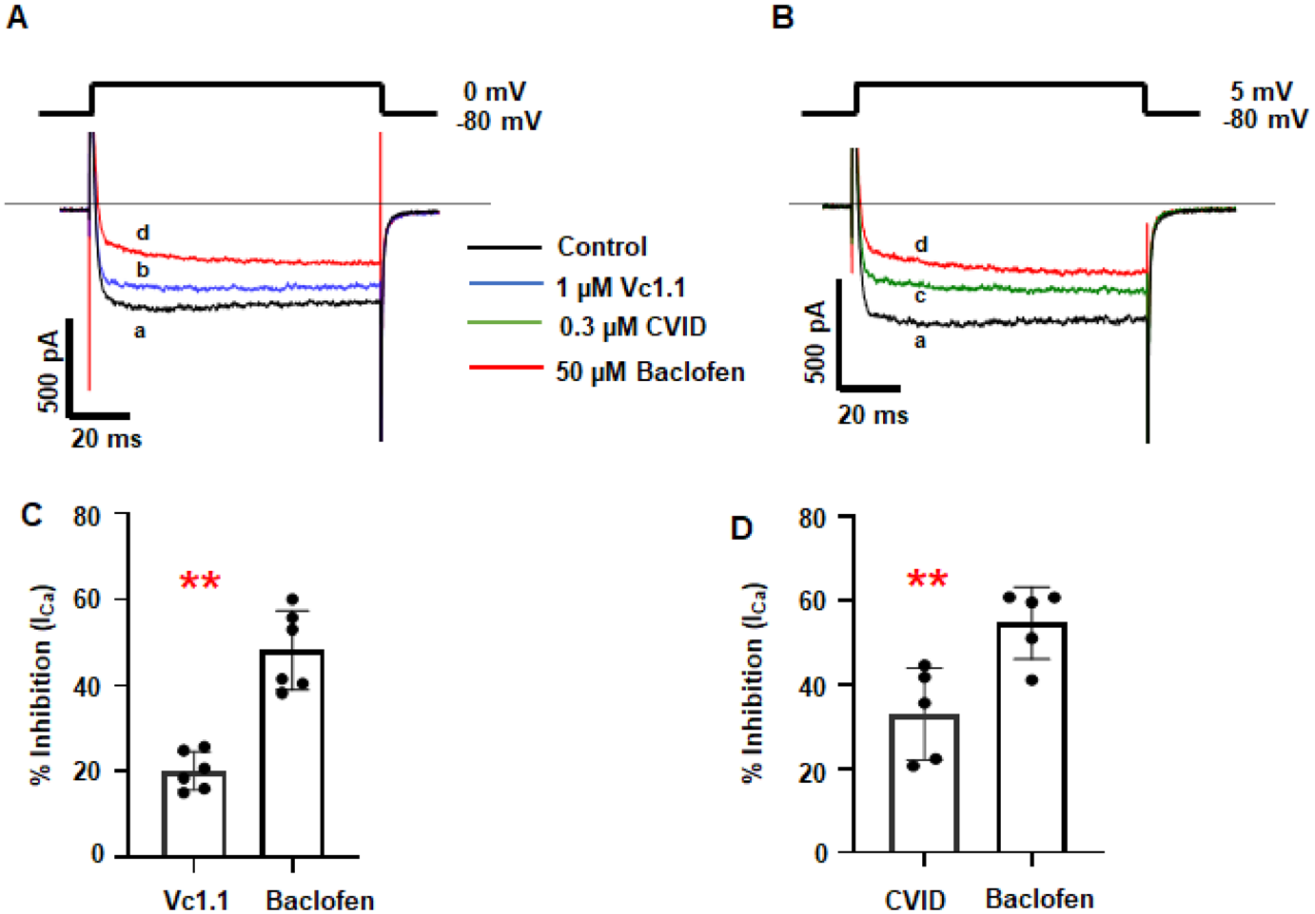
α‐Conotoxin Vc1.1 and GABA_B_R agonist baclofen inhibit high voltage‐activated (HVA) Ca^2+^ currents in H9^NGN2^‐induced sensory neurons (iSNs). (A, B) Representative depolarization‐activated Ca^2+^ currents (*I*_Ca_) recorded from iSNs in the absence (a, control) and presence of (b) 1 μM Vc1.1, (c) 0.3 μM ω‐conotoxin CVID, and (d) 50 μM baclofen. Panel A shows the inhibition of *I*_Ca_ by Vc1.1, whereas panel B shows the inhibition by CVID. (C, D) Bar graphs showing the effects of 1 μM Vc1.1 and 0.3 μM CVID compared to 50 μM baclofen on HVA Ca^2+^ current amplitude. Statistical analysis was performed using paired Mann–Whitney *U* tests. Data are presented as mean ± SD, **p = 0.0016 (*n* = 5–6 per each group).

Under our recording conditions, no inward Na^+^ current was observed in iSNs. α‐Ctx Vc1.1 (1 μM) robustly inhibited inward *I*_Ca_ in all neurons tested (*n* ≥ 6), with further inhibition observed upon application of 50 μM baclofen (Figure 6A,C). The contribution of N‐type Ca^2+^ channels to the whole‐cell *I*_Ca_ was evaluated by bath application of the selective N‐type Ca^2+^ channel inhibitor, ω‐conotoxin CVID. In the presence of 300 nM ω‐conotoxin CVID, *I*_Ca_ was reversibly inhibited by 33% ± 9% (Figure 6B,D). Bath application of the selective GABA_B_R antagonist CGP 55845 (1 μM) abolished the inhibition of *I*_Ca_ observed in the presence of 50 μM baclofen (Figure S2), indicating that the inhibition is mediated via GABA_B_R activation. Comparison of the inhibition of the baclofen‐sensitive *I*_Ca_ by both Vc1.1 and CVID is shown in the bar graph (*n* = 5–7, *p* = 0.0016) (Figure 6C,D).

**FIGURE 7:**
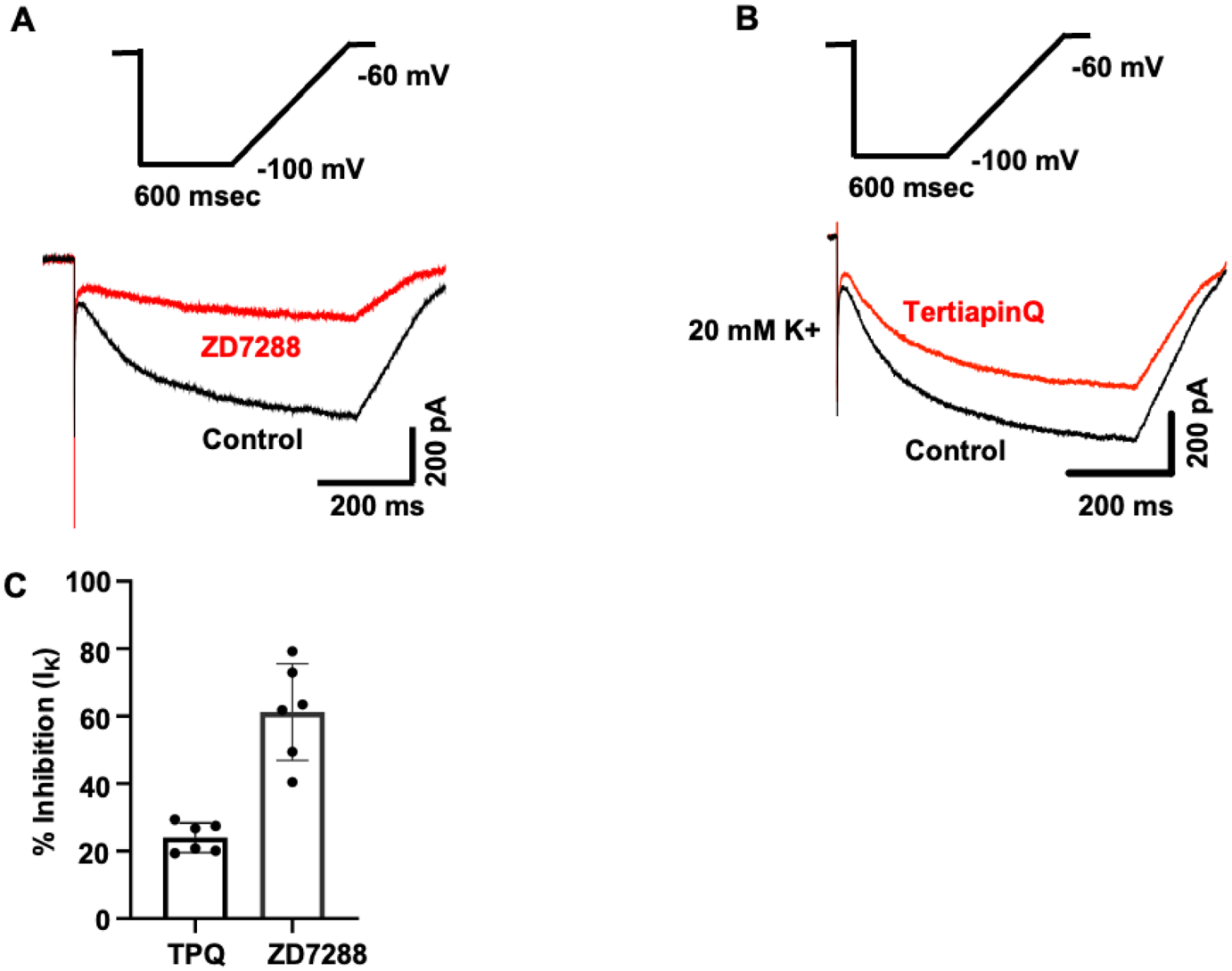
Representative hyperpolarisation‐activated currents recorded from H9^NGN2^‐induced sensory neurons (iSNs) in the presence of 20 mM extracellular K^+^ in response to a voltage‐clamp protocol ranging from −60 to −100 mV. Inward currents were measured in the presence of 10 μM ZD7288 (A) and 100 nM Tertiapin‐Q (B). (C) Bar graph comparing the effects of 100 nM TPQ and 10 μM ZD7288 on *I*_K_ inhibition. Data are presented as mean ± SD (*n* = 6 per group).

To confirm the functional presence of GIRK and HCN channels in iSNs, the effects of the specific inhibitors TPQ and ZD7288 were examined in response to hyperpolarising voltage steps. Using an elevated extracellular K^+^ concentration of 20 mM, a voltage step from −40 to −100 mV was applied, and the effects of the inhibitors were tested using bath superfusion. All cells showed robust, reversible inhibition of the hyperpolarisation‐activated inward current in response to 100 nM TPQ and 10 μM ZD7288 (*n* = 6; Figure 7A,B), confirming the functional presence of these channels in iSNs.

### 3.6 GABA_B_R Modulation of HCN Channels Expressed in HEK293T Cells

To further confirm the modulation of HCN channels by GABA_B_R activation, HEK293T cells expressing human GABA_B_Rs and HCN1/2 channels were exposed to either baclofen or α‐Ctx Vc1.1. In the presence of 100 μM baclofen and 1 μM Vc1.1, the hyperpolarisation‐activated inward current amplitude at −100 mV was potentiated by approximately 40% compared to control (*n* = 5–7; Figure S3A–C). The current–voltage relationship obtained with 1 μM Vc1.1 and 100 μM baclofen shifted the reversal potential to more negative membrane potentials and potentiated both inward and outward currents (Figure S3B). This potentiation of the hyperpolarisation‐activated inward current was inhibited by either ZD7288 (10 μM) or pre‐treatment of the transfected cells with PTX (0.1 μg/mL) for 24 h (Figure S4), indicating that the potentiation of the inward current by baclofen and Vc1.1 was mediated by G protein modulation of HCN channels.

## 4 Discussion

This study is the first to report GABA_B_R modulation of membrane excitability in hPSC‐derived sensory neurons by baclofen and α‐Ctx Vc1.1. To generate iSNs, hPSCs were first differentiated into NCC progenitors, which robustly expressed CD271 and SOX10. Subsequent NGN2 induction drove sensory differentiation of the NCCs. Characterisation of the resulting mixed population of iSNs via immunofluorescence staining and transcriptomic analysis revealed upregulated expression of key DRG sensory lineage markers (*BRN3A*, *ISL1,* and *PRPH*) compared to hPSC. These iSNs further possessed the molecular machinery required for membrane excitability, including the expression of GABA_B_R and Ca_V_2.2, GIRK1, and HCN ion channels (Hulme et al. 2020).

Hyper‐excitability and ectopic firing are characteristic responses of sensory neurons to nerve injury and chronic pain (Amir, Kocsis, and Devor 2005; Berta et al. 2017). The functional characterisation of iSNs included electrophysiological assessment of GABA_B_R activity and neuronal membrane excitability. GABA_B_R activators baclofen and α‐Ctx Vc1.1 significantly reduced membrane excitability of iSNs by hyperpolarising the RMP and increasing the rheobase for AP firing. In voltage‐clamp mode, both baclofen and Vc1.1 inhibited HVA Ca^2+^ channel currents, which was attenuated by the selective GABA_B_R antagonist, CGP 55845. Furthermore, baclofen inhibition of GABA_B_R‐coupled HVA N‐type calcium channels was confirmed using ω‐conotoxin CVID, a selective N‐type (Cav2.2) channel inhibitor.

Although several studies have demonstrated a role for GABA_B_R in modulating inwardly rectifying K^+^ channels in rodent DRG neurons (Bony et al. 2022; Gao et al. 2007; Marker et al. 2006), neither baclofen nor Vc1.1 significantly potentiated K^+^ currents in human sensory neurons, suggesting a limited interaction between GABA_B_R and these channels in humans. However, when iSNs were exposed to Tertiapin‐Q and ZD7288, specific inhibitors of GIRK and HCN channels, respectively, the hyperpolarisation‐activated inwardly rectifying K^+^ current in these neurons was reduced. These results confirm the presence of functional GIRK1/2 and HCN channels in iSNs. However, no evidence of direct coupling between GABA_B_R and HCN channels was observed in iSNs, despite proteomic studies identifying HCN channels as signalling GABA_B_R effectors in other models (Schwenk et al. 2016). In HEK293T cells co‐expressing human GABA_B_R and HCN1/2 channels, both baclofen and Vc1.1 potentiated hyperpolarisation‐activated inward currents, which were inhibited by ZD7288 or by pre‐treating the transfected cells with PTX. This finding suggests a mechanism mediated by PTX‐sensitive Gi/o proteins, but further studies are needed to clarify whether similar interactions occur in iSNs.

The effects of baclofen and Vc1.1 on iSN excitability provide a foundation for exploring GABA_B_R‐mediated signalling in human sensory neurons. Extensive research on GABA_B_R‐mediated signalling has often utilised rodent tissues or transfected cell lines (Fritzius et al. 2022; Rose and Wickman 2022). While these models have been invaluable, recent studies highlight the fundamental differences between human sensory tissues and those of model organisms, with distinct variances in neuronal subtypes and ion channel expression (Labau et al. 2022; Rostock et al. 2018; Schwaid et al. 2018).

The development of hPSC‐derived sensory neurons overcomes the physiological inconsistencies of animal tissues and accessibility issues of primary human DRG. Using transcription factor‐based differentiation systems, such as NGN2 for sensory neurons, enables reproducible generation of human‐like sensory neurons (Hulme et al. 2020; Nickolls et al. 2020; Plumbly et al. 2022; Schrenk‐Siemens et al. 2015). Comparison of iSN transcriptomic profiles with a single‐cell nucleus RNA sequencing dataset of human DRG (Jung et al. 2023) revealed positive correlation in the expression of Ca^2+^ channels and GABA_B_Rs, though no correlation in GIRK or HCN channel expression. Protein‐level validation confirmed the presence of key ion channels and GABA_B_Rs, supporting the relevance of iSNs as a model system.

In conclusion, this study provides novel insights into GABA_B_R modulation of membrane excitability in human iSNs. By characterising the effects of GABA_B_R activators baclofen and α‐Ctx Vc1.1, we established a platform for investigating human‐specific GABA_B_R signalling. These findings highlight the utility of hPSC‐derived iSNs in bridging the gap between animal studies and human tissue, paving the way for continued research into novel analgesic compounds. Future studies should focus on nociceptive neuron subtypes to further explore pain mechanisms and develop effective pain therapeutics.

## Author Contributions

**Mitchell St Clair‐Glover:** data curation, formal analysis, investigation, methodology, validation, writing – original draft, writing – review and editing, visualization. **Arsalan Yousuf:** data curation, formal analysis, investigation, methodology, validation, writing – original draft, writing – review and editing, visualization. **Dominic Kaul:** data curation, formal analysis, investigation, validation, writing – original draft, writing – review and editing, visualization. **Mirella Dottori:** conceptualization, funding acquisition, methodology, project administration, resources, supervision, writing – review and editing. **David J. Adams:** conceptualization, project administration, funding acquisition, writing – review and editing, resources, methodology, supervision.

## Conflicts of Interest

The authors declare no conflicts of interest.

### Peer Review

The peer review history for this article is available at https://www.webofscience.com/api/gateway/wos/peer‐review/10.1111/jnc.70004.

## Supporting information


Data S1.

## Data Availability

The data that support the findings of this study are available from the corresponding authors upon request. The bulk RNAseq data used in this study have been deposited at the Gene Expression Omnibus (GEO) (https://www.ncbi.nlm.nih.gov/geo/) with the accession number GEO: GSE282901 and are publicly available as the date of publication.
