# Supplementary material for: GABAB Receptor Modulation of Membrane Excitability in Human Pluripotent Stem Cell‐Derived Sensory Neurons by Baclofen and α‐Conotoxin Vc1.1
Source: J Neurochem. 2025 Jan 27;169(1):e70004. doi: 10.1111/jnc.70004 (PMC11773314; doi:10.1111/jnc.70004)
Supplement: Supplementary file 1 — Data S1. [file JNC-169-0-s001.pdf]

SUPPORTING INFORMATION

GABA$_B$ receptor modulation of membrane excitability in human pluripotent stem cell-derived sensory neurons by baclofen and α-conotoxin Vc1.1

Mitchell St Clair-Glover[1,¶], Arsalan Yousuf[1,2,¶], Dominic Kaul[1], Mirella Dottori[1,*], and David J. Adams[1,*]

[1] Molecular Horizons, Faculty of Science, Medicine and Health, University of Wollongong, Wollongong, NSW 2522 Australia.

[2] Sydney Pharmacy School, Faculty of Medicine and Health, The University of Sydney, NSW 2050 Australia.

[¶]Co-first authors

*Correspondence: mdottori@uow.edu.au; djadams@uow.edu.au

SUPPLEMENTARY FIGURES

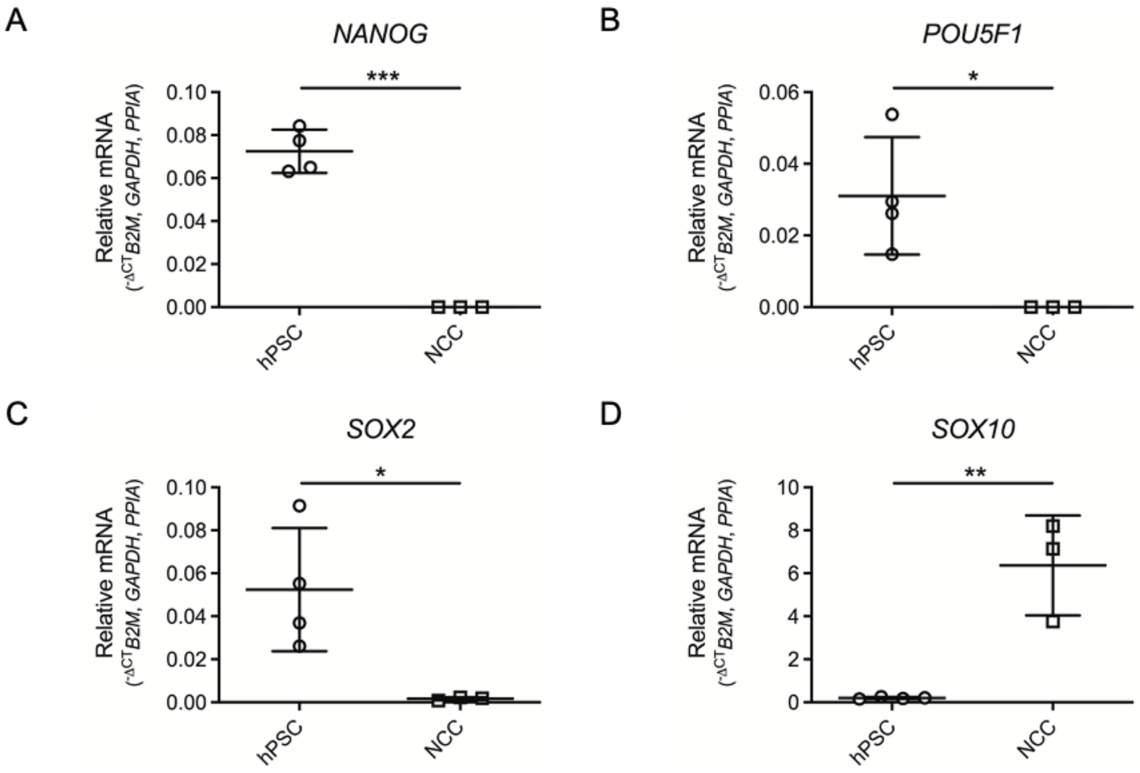

Figure S1. Differentiation of H9^{NGN2} human pluripotent stem cells (hPSC) to neural crest cells (NCC) is associated with downregulated expression of pluripotency markers. (**A-D**) Fold change in mRNA expression of pluripotency genes *NANOG* **(A)**, *POU5F1* **(B)**, and *SOX2* **(C)**, as well as the NCC marker *SOX10* **(D)** in H9^{NGN2}-hPSCs and NCCs. Expression levels were normalized to housekeeping genes *B2M*, *PPIA*, and *GAPDH* (n = 3 - 4 biological replicates). An unpaired t-test was used to assess significant differences in means, or a one-sample t-test was used where expression was below the detection threshold. Data are presented as mean ± SD, with significance indicated as ***p < 0.001, **p < 0.005, *p < 0.05.

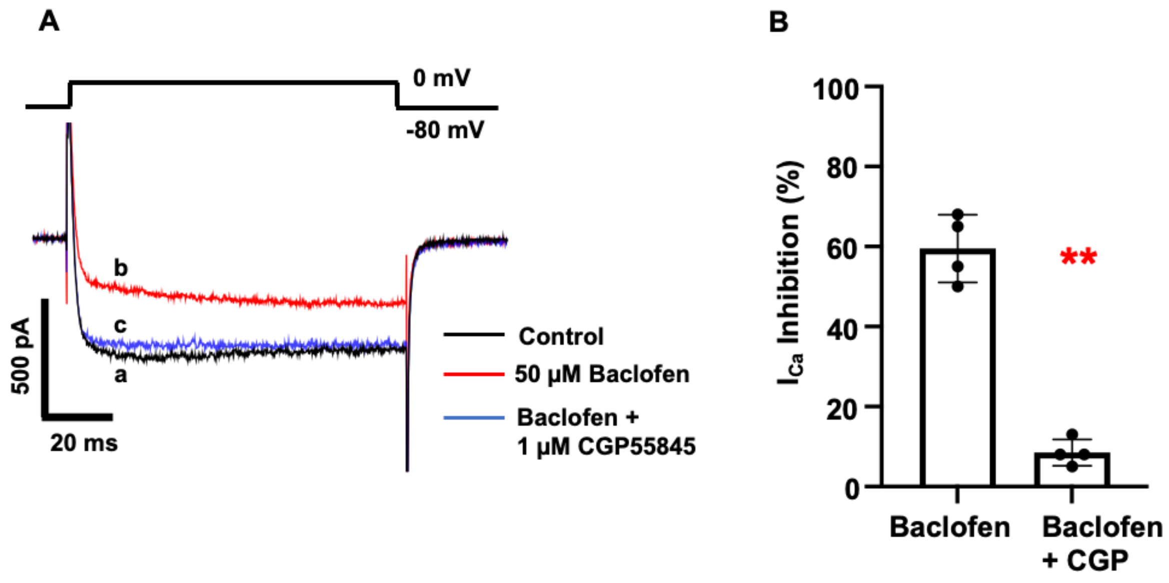

Figure S2. (A) Superimposed depolarization-activated HVA Ca^{2+} currents in H9[NGN2]-induced sensory neurons (iSNs) recorded in the absence (**a**, black) and presence (**b**, red) of the selective $GABA_BR$ agonist, baclofen (50 µM). The $GABA_BR$ antagonist CGP 55845 (1 µM) (**c**, blue) completely antagonized the effect of baclofen. The holding potential was –80 mV, and the test potential 0 mV. **(B)** Bar graph comparing HVA Ca^{2+} currents (I_{Ca}) in iSNs in response to 50 µM baclofen, in the absence and presence of the CGP55845 (1 µM). Data were analysed using a paired t-test and are presented as mean ± SD (**p = 0.0021, n = 4 per group).

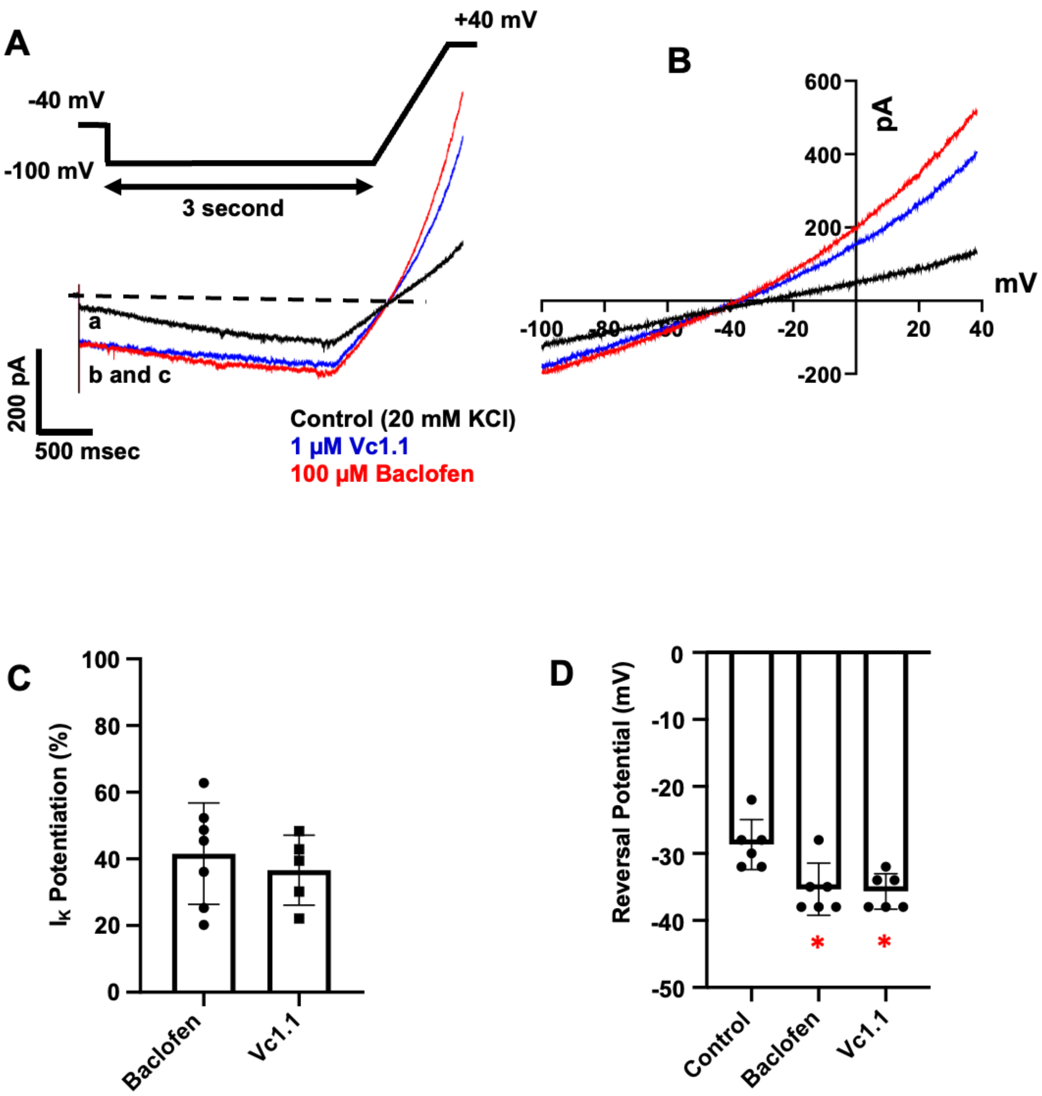

Figure S3. Effects of baclofen and α-conotoxin Vc1.1 on hyperpolarization-activated currents in HEK293T cells co-expressing human $GABA_BR$ and HCN1/2 channels. (**A**) Representative whole-cell patch clamp recording of hyperpolarization-activated currents in HEK293T cells with 20 mM extracellular K^+, recorded in the absence (**a**, control) and presence of 1 µM Vc1.1 (**b**) and 100 µM baclofen (**c**). (**B**) Current-voltage (I-V) relationship showing responses in the absence (control) and presence of 1 µM Vc1.1 (blue) and 100 µM baclofen (red). Both Vc1.1 and baclofen shift the reversal potential to more negative membrane potentials and potentiate inward and outward currents. (**C, D**) Bar graph comparing the effects of 100 µM baclofen and 1 µM α-Ctx Vc1.1 on the potentiation of the hyperpolarization-activated inward K^+ current at −100 mV **(C)** and on changes in reversal potential of the I-V relationship **(D)**. Data for I_K potentiation were analysed using a paired t-test and for reversal potential using Kruskal-Wallis test, Dunns multiple comparison (*p = 0.0301, n = 5-7 per group). All data are presented as mean ± SD.

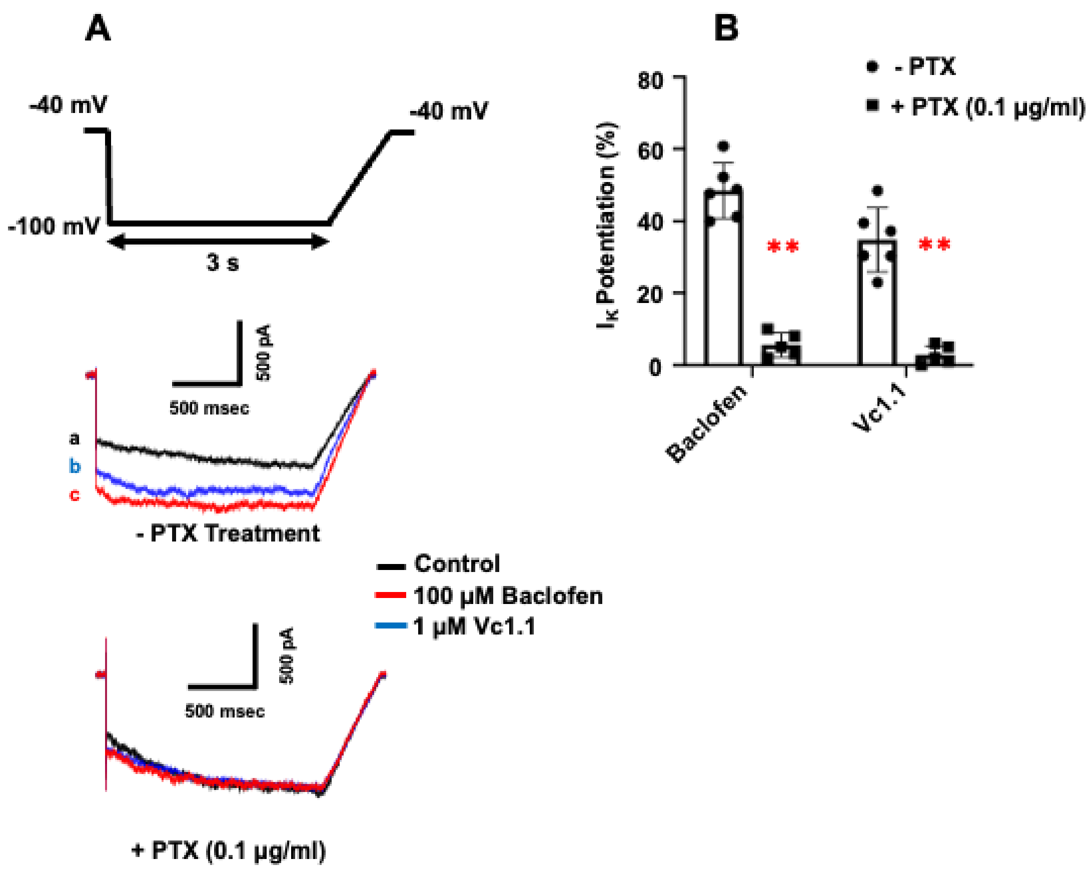

Figure S4. Effect of Pertussis toxin (PTX) on baclofen and α-conotoxin Vc1.1-induced potentiation of hyperpolarization-activated currents in HEK293T cells co-transfected with human $GABA_BR$ and HCN1/2 subunits. (**A**) Representative whole-cell patch clamp recordings of hyperpolarization-activated inward currents in HEK293T cells with 20 mM extracellular K^+, obtained in the absence (**a**, control) and presence of 1 µM Vc1.1 (**b**) and 100 µM baclofen (**c**). Recordings were obtained from transfected HEK293T cells without (–PTX) and after pretreatment with 0.1 µg/mL PTX (+PTX). (**B**) Bar graph comparing the effects of 100 µM baclofen and 1 µM α-Ctx Vc1.1 on hyperpolarization-activated inward currents at –100 mV, both without PTX and following PTX pretreatment. Data were analysed using Tukey's multiple comparison test and are presented as mean ± SD (***p* < 0.01, n = 6 per group).
